# Squamocin as the Principal Toxic Constituent of *Annona squamosa* Seeds: Toxicity-Guided Isolation, Developmental and Neurotoxicity, and Metabolic Perturbations

**DOI:** 10.3390/ijms27156802

**Published:** 2026-07-29

**Authors:** Chenyuan Qi, Lu Zhang, Xue Li, Mengling Zhang, Disheng Wang, Qiang Zhang

**Affiliations:** Shaanxi Key Laboratory of Natural Products & Chemical Biology, College of Chemistry & Pharmacy, Northwest A&F University, Yangling 712100, China; qichenyuan@nwafu.edu.cn (C.Q.); zhanglu622@nwafu.edu.cn (L.Z.); lixue56@nwafu.edu.cn (X.L.); 2024051708@nwafu.edu.cn (M.Z.); 2020011944@nwafu.edu.cn (D.W.)

**Keywords:** sweetsop seeds, toxicity-guided isolation, component, squamocin, developmental toxicity, metabolic intervention

## Abstract

The seeds of *Annona squamosa* are widely used in traditional medicine and food, yet their safety profile remains unclear and the principal toxic constituent is unknown. This study aimed to identify the main toxic constituent and elucidate its toxicological mechanisms. A toxicity-guided isolation strategy using zebrafish larvae was employed, combined with morphological, behavioral, biochemical, metabolomic, and transcriptional analyses. Bioassay-directed fractionation identified squamocin, an annonaceous acetogenin, as the principal toxic constituent—the first reported acute toxicant from *A. squamosa*. Squamocin exerted potent multi-organ toxicity at nanomolar concentrations (${\mathrm{LC}}_{10}$ = 62 nM; ${\mathrm{LC}}_{50}$ = 75 nM), including lethality, developmental teratogenicity, and craniofacial cartilage dysplasia, without substantial growth retardation. Neurotoxicity was dose-dependent: locomotor suppression at 20 nM and motor incoordination at 40 nM. Metabolomics revealed perturbations in amino acid and glycan metabolism, and transcriptional analysis confirmed altered expression of stress-related genes (*pipox*, *myd88*, and *rigi*). These findings establish squamocin as the major toxicant in *A. squamosa* seeds and provide a mechanistic framework for its broad toxicological spectrum, informing safer use of this material.

## 1. Introduction

*Annona squamosa* L. (Annonaceae), commonly known as sugar apple or custard apple, is a tropical fruit tree native to the West Indies, Central and South America, and widely cultivated across Asia, Africa, and Egypt [1,2]. Its edible fruit pulp is appreciated for a well-balanced nutrient profile rich in carbohydrates, proteins, amino acids, vitamins (especially vitamin C), minerals, and dietary fibers [1]. Beyond direct consumption, the pulp is used as a flavour enhancer in ice cream, yogurt, smoothies, and beverages [1].

While the fruit pulp is the primary edible portion, the seeds are usually discarded as waste during processing. However, phytochemical investigations have revealed that the seeds concentrate substantial quantities of bioactive secondary metabolites, particularly acetogenins (ACGs), at levels often higher than those found in the pulp [1,3]. This stark difference between the nutritional value of the pulp and the chemical richness of the seeds underscores the necessity of evaluating the safety profile of the seed material.

Chemical investigations of *A. squamosa* seeds have documented a diverse array of secondary metabolites. The major constituents include annonaceous ACGs, alkaloids, diterpenes, and cyclopeptides, among which ACGs and cyclopeptides are reported to be concentrated mainly in the seeds [1,3]. ACGs are a unique class of $C_{35}$/$C_{37}$ long-chain fatty acid derivatives terminated by an α,β-unsaturated γ-lactone ring and often substituted with tetrahydrofuran rings, epoxides, hydroxyl, or carbonyl groups along the aliphatic chain [3].

Recent isolation studies have continued to yield new ACGs from *A. squamosa* seeds; for example, annotemoyins L, Y, and X were identified and exhibited promising cytotoxicity against multidrug-resistant cancer cell lines such as SMMC-7721/ADR, A549/T, and MCF-7/ADR [4]. In addition, seed extracts have demonstrated free-radical-scavenging capacity and inhibitory effects against epidermal growth factor receptor kinase, leading to suppressed proliferation of MCF7 breast cancer cells [5]. These findings highlight the pharmacological potential of *A. squamosa* seeds, yet they also raise concerns because crude seed extracts have simultaneously shown genotoxic and toxic properties in experimental models [6].

Parallel to these promising pharmacological properties, the toxicological profile of annonacin has become an area of intense concern. Epidemiological and experimental evidence has linked chronic consumption of Annonaceae products containing annonacin to Caribbean atypical parkinsonism (CAP), a levodopa-resistant neurodegenerative disorder [7]. Post-mortem histopathological analyses of CAP patients revealed complex tau/α-synuclein copathology, and biophysical assays demonstrated that annonacin accelerates α-synuclein aggregation and cross-seeds tau aggregation [7].

At the cellular level, annonacin functions as a potent mitochondrial complex I inhibitor, causing energetic impairment, tau hyperphosphorylation, and neurite degeneration in dopaminergic neurons, thereby mimicking tauopathy-like features [8]. Toxicity evaluations of acetogenin-rich seed extracts from *Annona muricata* further corroborated these hazards: the extracts reduced the mitotic index in *Allium cepa* meristematic cells, caused cell necrosis, and displayed acute toxicity toward *Artemia salina* [6].

However, current studies have largely focused on annonacin or a few known ACGs, while other toxic constituents in *A. squamosa* seeds remain unexplored. Moreover, the prevailing mechanistic view—mitochondrial complex I inhibition and tau/α-synuclein proteinopathy—may not fully explain all adverse effects, as alternative toxicological endpoints (e.g., metabolic disruption, inflammation) have not been systematically investigated at the single-compound level. Prior assessments have also relied predominantly on crude extracts or fractions [6], precluding precise identification of toxic entities and rigorous mechanistic interpretation. An integrated strategy combining toxicity-guided isolation with metabolomic and molecular verification is therefore essential to move beyond the current “annonacin-centric” understanding.

In light of these considerations, the present study was designed to investigate the toxic constituents of *A. squamosa* seeds through a toxicity-guided isolation strategy. Herein, we report the targeted identification of a toxic annonaceous acetogenin from the seeds and characterize its toxicological features.

## 2. Results

### 2.1. Toxicity-Guided Isolation and Structural Characterization

**Figure 1:**
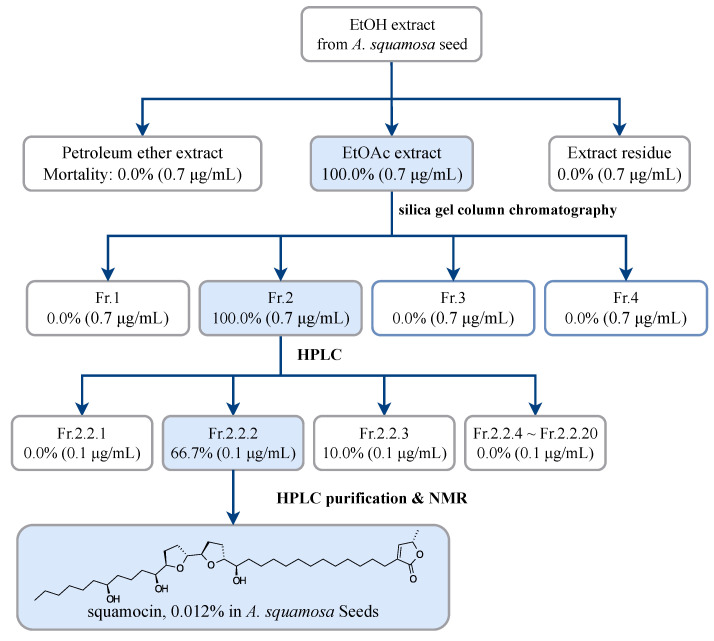
Toxicity-guided isolation and the chemical structure of squamocin.

The dried seeds of *A. squamosa* were successively extracted and partitioned to trace the toxic constituent as shown in Figure 1. Ethanol extraction followed by solvent partitioning yielded petroleum ether, ethyl acetate (EtOAc), and aqueous residues. At a test concentration of 0.7 µg/mL, the EtOAc fraction induced 100.0% mortality, whereas the petroleum ether fraction and the aqueous residue showed no lethality, indicating that the toxic component was enriched in the EtOAc-soluble portion. The EtOAc fraction was further separated by silica gel column chromatography into four subfractions (Fr.1–Fr.4). Toxicity assessment at 0.7 µg/mL revealed that Fr.2 was exclusively responsible for the toxic effect (100.0% mortality), while Fr.1, Fr.3, and Fr.4 were non-toxic. Subsequent HPLC fractionation of Fr.2 gave Fr.2.2.1–Fr.2.2.4, which were evaluated at 0.1 µg/mL. Among them, Fr.2.2.2 showed the highest toxicity (66.7% mortality), followed by Fr.2.2.3 (10.0% mortality), whereas Fr.2.2.1 and Fr.2.2.4 caused no mortality. Final HPLC purification, NMR and HR-MS analysis (Figure S1 in Supplementary Material) of Fr.2.2.2 identified the toxic compound as squamocin [9], which accounted for 0.012% of the fresh seeds.

Subsequently, the toxic effects of squamocin were evaluated with respect to developmental toxicity, cartilage development, neurological function, and metabolic perturbation.

### 2.2. Squamocin-Induced Developmental Toxicity

**Figure 2:**
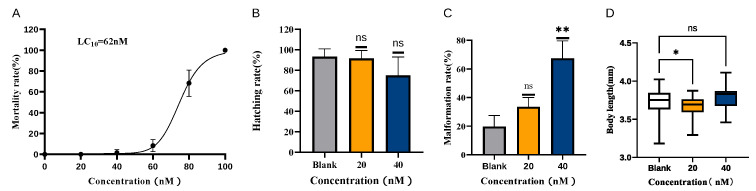
Zebrafish toxicity assessment of squamocin in zebrafish larvae: (**A**) concentration-dependent survival rate following 48 h exposure; (**B**) hatching rate; (**C**) malformation rate; and (**D**) body length. For (**A**–**C**), 3 biological replicates, 20 larvae per replicate for (**D**), *n* = 9. Values are expressed as the mean ± SD. Significant differences versus the control group are denoted as * p<0.05 and ** p<0.01.

The developmental toxicity was evaluated by exposing zebrafish larvae to squamocin and monitoring mortality, hatching rate, malformation rate, and body length. To evaluate the acute toxicity of squamocin, zebrafish larvae were exposed to a series of concentrations for 48 h (Figure 2). As shown in Figure 2A, larval mortality increased in a concentration-dependent manner. At low concentrations (20 and 40 nM), mortality remained negligible. The ${\mathrm{LC}}_{10}$ value was determined to be 62 nM. At concentrations exceeding 62 nM, mortality increased sharply, culminating in 100.0% (all dead) mortality at 100 nM. Collectively, these results demonstrate that squamocin is an ultra-potent toxicant, with an ${\mathrm{LC}}_{10}$ of 62 nM and an ${\mathrm{LC}}_{50}$ of 75 nM.

The effects of squamocin on embryonic hatching are summarized in Figure 2B. Exposure to 20 and 40 nM squamocin reduced the hatching rate compared with the blank control group. Quantitatively, the hatching rates were 91.7% at 20 nM and 75.0% at 40 nM. Although the mean showed a downward trend, it did not reach statistical significance.

The teratogenic potential of squamocin was further assessed by quantifying developmental malformation rates (Figure 2C). While the 20 nM group showed no significant increase in malformations, the 40 nM group exhibited a significant increase (p<0.01). The main malformations included spinal curvature, pericardial edema, and delayed yolk sac absorption. These malformations increased in a dose-dependent manner.

Larval body length was measured to evaluate overall growth inhibition. As depicted in Figure 2D, exposure to 20 nM squamocin resulted in a slight but significant reduction in larval body length (p<0.05). In contrast, no significant change in body length was observed at 40 nM.

### 2.3. Squamocin-Induced Alterations in Cartilage Development

**Figure 3:**
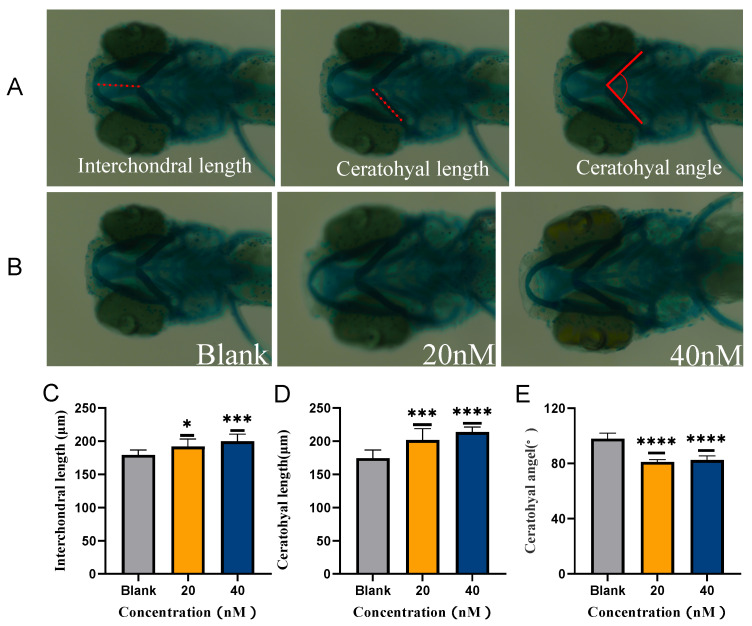
Toxcity of squamocin on craniofacial cartilage development in zebrafish larvae. (**A**) Measurement scheme established for evaluating craniofacial structures. (**B**) Dorsal view of the head region in zebrafish larvae stained with Alcian blue. (**C**) Distance between Meckel’s cartilage and ceratohyal after squamocin exposure. (**D**) Length of the ceratohyal altered by squamocin. (**E**) Angle of the ceratohyal affected by squamocin. Data are presented as mean ± SD, n=9; ns, not significant; * p<0.05, *** p<0.001, **** p<0.0001.

To assess whether squamocin exposure impairs craniofacial morphogenesis, zebrafish larvae were treated with squamocin and subjected to Alcian blue staining to visualize cartilage structures. As illustrated in Figure 3A, a standardized measurement scheme was established to quantify three key craniofacial parameters: the interchondral distance between Meckel’s cartilage and the ceratohyal, the length of the ceratohyal, and the angle formed by the ceratohyal. Representative dorsal-view images of the head region are presented in Figure 3B. Compared with the blank control, larvae exposed to 20 nM and 40 nM squamocin displayed conspicuous morphological abnormalities in craniofacial cartilages, including altered spatial relationships among skeletal elements and disrupted cranial base architecture.

As shown in Figure 3C, the distance between Meckel’s cartilage and the ceratohyal was significantly shortened in both the 20 nM and 40 nM exposure groups relative to the blank control group, and the magnitude of reduction was enhanced in a dose-dependent manner with increasing exposure concentration. The length of the ceratohyal was significantly reduced in both the 20 nM and 40 nM exposure groups (Figure 3D). The high-concentration group exhibited a more prominent short-cartilage dysmorphic phenotype, demonstrating a typical dose–effect relationship. As depicted in Figure 3E, the ceratohyal angle was slightly increased in the 20 nM exposure group and became significantly enlarged in the 40 nM exposure group, accompanied by disorganized cartilage arrangement, aberrant spatial polarity, and disrupted craniofacial symmetry.

### 2.4. Squamocin-Induced Alterations in Neurological Function

**Figure 4:**
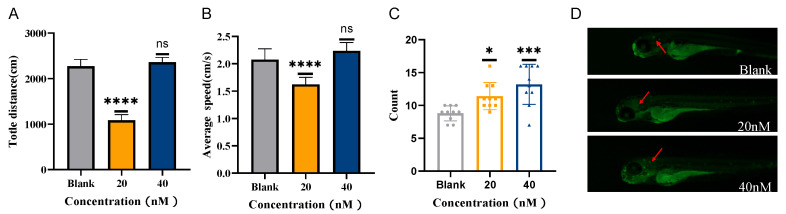
Neurotoxic effects of squamocin on zebrafish larvae. (**A**) Total locomotor distance of zebrafish larvae after squamocin exposure. (**B**) Behavioral analysis of average swimming speed. (**C**) Apoptosis of neural cells in the brain of zebrafish larvae. (**D**) Fluorescence micrograph showing apoptotic neural cells; red arrows indicate apoptotic neural cells. Data are presented as mean ± SD, n=9; ns, not significant; * p<0.05, *** p<0.001, **** p<0.0001.

To investigate the neurotoxic potential of squamocin, zebrafish larvae were exposed to squamocin and subsequently evaluated for locomotor activity and neuronal apoptosis. As shown in Figure 4A, the total locomotor distance of larvae in the 20 nM exposure group was markedly reduced compared with the blank control group (p<0.0001), whereas the 40 nM exposure group showed only a marginal change in total movement distance.

Consistent with the locomotor distance data, the average swimming speed of larvae in the 20 nM exposure group was markedly significantly decreased relative to the blank control (p<0.0001), while no substantial change was observed in the 40 nM group (Figure 4B).

Quantitative analysis of neuronal apoptosis in the larval brain revealed that, compared with the blank control group, both the 20 nM and 40 nM exposure groups exhibited a significant increase in the apoptotic rate of brain neurons, displaying a clear dose-dependent trend (Figure 4C).

This dose-dependent increase in neuronal apoptosis was further corroborated by fluorescence microscopy (Figure 4D). Representative images showed that the number of apoptotic neural cells in the brain region was markedly elevated in both the 20 nM and 40 nM exposure groups compared with the blank control, with apoptotic cells clearly indicated by red arrows.

**Figure 5:**
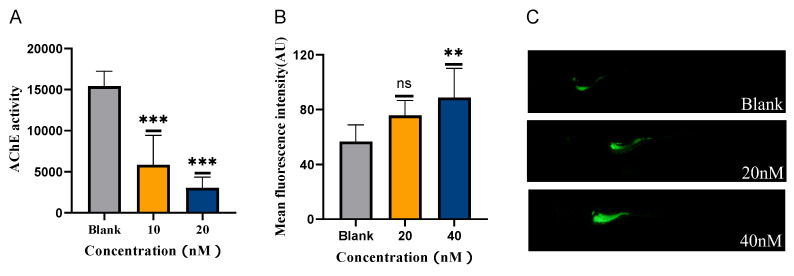
Effects of squamocin on AChE activity and ROS levels in zebrafish larvae. (**A**) AChE activity in the brain of zebrafish larvae after squamocin exposure. (**B**) ROS levels in the brain of zebrafish larvae following squamocin exposure. (**C**) The green fluorescence signal in the zebrafish larva. Data are presented as mean ± SD, n=9; ns, not significant; ** p<0.01, *** p<0.001.

To elucidate the biochemical mechanisms underlying the observed neurotoxic effects, we next examined the alterations in AChE activity and ROS levels in squamocin-exposed zebrafish larvae. As shown in Figure 5A, AChE activity was significantly inhibited in both the 10 nM and 20 nM exposure groups compared with the blank control group (p<0.001), with the magnitude of inhibition intensifying as the concentration increased.

Quantitative analysis of ROS levels revealed that the mean fluorescence intensity was elevated in a dose-dependent manner, and the increase reached statistical significance in the 40 nM exposure group (p<0.01) compared with the blank control, whereas the 20 nM group showed an upward trend without reaching significance (Figure 5B). This dose-dependent accumulation of ROS was further corroborated by fluorescence microscopy images, in which the green fluorescence signal in the larval body was markedly intensified in both the 20 nM and 40 nM exposure groups compared with the blank control (Figure 5C).

### 2.5. Metabolic Perturbations Induced by Squamocin Exposure

**Figure 6:**
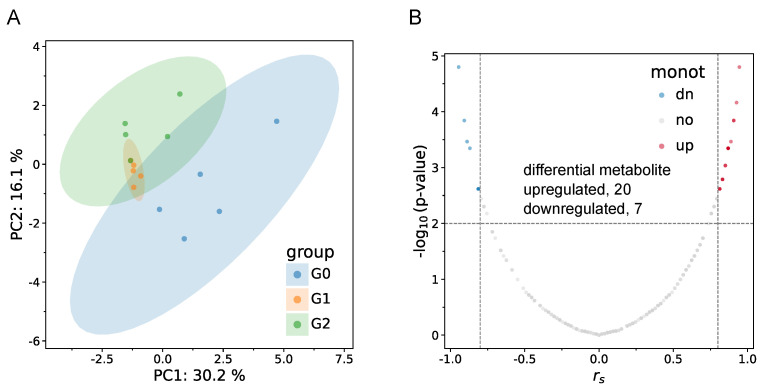
Metabolic perturbations induced by squamocin exposure in zebrafish larvae. (**A**) PCA score plot showing the separation of metabolic profiles between squamocin-exposed and control groups. (**B**) Spearman correlation analysis of significantly altered metabolites (G0→G1→G2). G0, blank control group; G1, 10 nM squamocin exposure group; G2, 20 nM squamocin exposure group.

To investigate the metabolic impact of squamocin exposure, untargeted metabolomic analysis was performed on zebrafish larvae. As shown in Figure 6A, PCA revealed a clear separation between the control group (G0) and squamocin-exposed groups (G1 and G2) along PC1, which accounted for 30.2% of the total variance, indicating substantial metabolic perturbations induced by squamocin. Spearman correlation analysis further identified 27 significantly altered metabolites, among which 20 were upregulated and 7 were downregulated in a concentration-dependent manner (G0 → G1 → G2) (Figure 6B).

**Figure 7:**
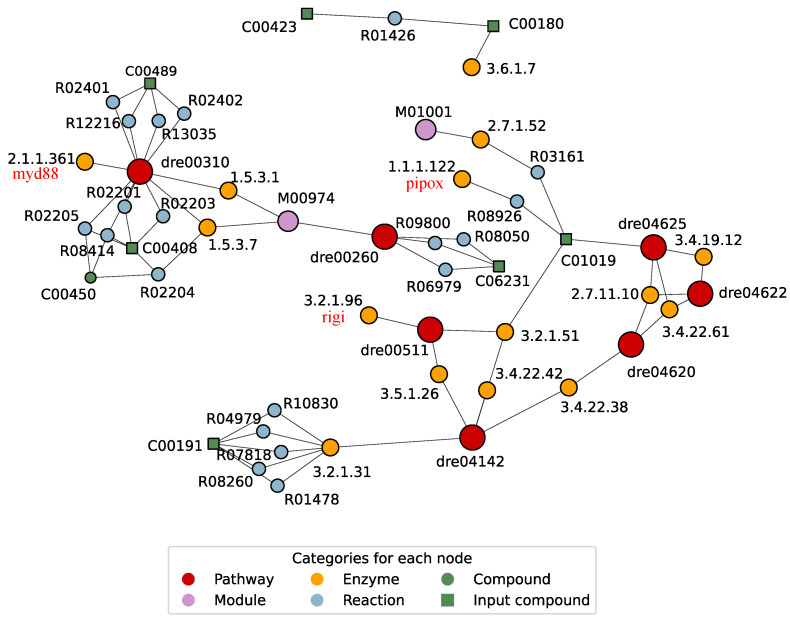
Metabolic pathway enrichment network based on differentially expressed metabolites. Nodes represent enriched pathways, functional modules, and differential metabolites; edges indicate their relationships. Genes encoding enzymes EC 1.1.1.122, EC 2.1.1.361, and EC 3.2.1.96 showed significantly altered expression levels in qRT-PCR validation (Figure 8). See Table S1 in the Supplementary Material for detailed information on all nodes.

To elucidate the biological implications of these metabolic changes, pathway enrichment analysis was performed using the 27 differential metabolites. This analysis revealed multiple significantly associated metabolic pathways and modules (as shown in Figure 7 and Table S1 in Supplementary Material), including lysine degradation (dre00310), glycan degradation (dre00511), and C-type lectin receptor signaling pathway (dre04625), as well as metabolic modules such as betaine metabolism (M00974). The network relationships among these enriched pathways, modules, and the differential metabolites are visualized in Figure 7.

**Figure 8:**
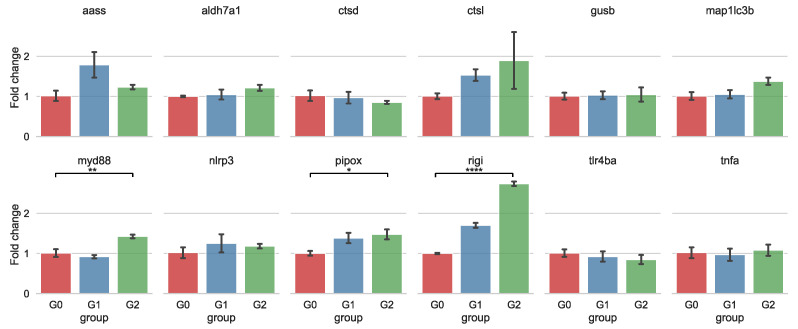
Expression of key genes in squamocin-exposed zebrafish larvae; * p<0.05, ** p<0.01, **** p<0.001.

To validate the pathways identified by enrichment analysis, the catalytic enzymes associated with the enriched pathways were extracted from the FELLA network, and their corresponding genes were subsequently selected for quantitative real-time PCR (qRT-PCR) verification. As shown in Figure 8, the mRNA expression levels of *rigi*, *pipox*, and *myd88* were all significantly elevated in squamocin-exposed zebrafish larvae compared with the blank control group. Notably, *rigi* exhibited the most pronounced upregulation in a concentration-dependent manner.

## 3. Discussion

### 3.1. Zebrafish Larvae as an Ideal Toxicity-Guided Tracking Model

Zebrafish larvae offer distinct advantages for toxicity-guided isolation of natural products, including embryonic transparency, high genetic homology to humans, and minimal compound requirements with rapid readouts. These features make them well suited to iterative fractionation workflows. The effectiveness of this strategy has been demonstrated by previous identification of toxic natural products using zebrafish as an indicator species [10]. The model has also been validated for evaluating nephrotoxicity within complex traditional Chinese medicine systems [11], and transgenic lines enable real-time tracing of neurotoxicity and cardiotoxicity [12,13].

In the present study, we employed zebrafish larvae to guide the fractionation of *A. squamosa* seeds. At 0.7 µg/mL, only the EtOAc fraction induced complete mortality (100.0%), indicating that the toxic component was enriched in this soluble portion. Iterative chromatographic separation coupled with toxicity assessment at 0.1 µg/mL on zebrafish larvae guided the isolation of squamocin as the most toxic constituent. To our knowledge, this represents the first identification of an acute toxic constituent from *A. squamosa*. Our results also demonstrate that zebrafish larvae can be conveniently employed as a toxicity-tracking model for guiding the fractionation of natural products.

### 3.2. Squamocin Induces Developmental Teratogenicity and Craniofacial Cartilage Dysplasia

Squamocin exhibits broad-spectrum, potent antitumor activity with a low propensity for drug resistance, acting through the mitochondria–endoplasmic reticulum–ubiquitination axis. In a 4T1 orthotopic breast cancer mouse model, the compound significantly suppressed primary tumor growth and prolonged the survival of tumor-bearing mice, demonstrating superior efficacy compared with paclitaxel injection [14]. Mechanistically, squamocin specifically targets heat shock protein 90α (HSP90α), thereby inhibiting mitochondrial respiratory chain complex I and leading to reduced ATP production. Concurrently, it induces endoplasmic reticulum stress and the unfolded protein response, activates the UBA6–UBE2Z–FBXW7 ubiquitination cascade, and promotes the ubiquitin-dependent degradation of oncoproteins EZH2 and MYC [15].

Despite the remarkable antitumor efficacy reported for squamocin, our toxicity-guided investigation of *A. squamosa* seeds identified this compound as a potent toxicant and a principal contributor to the overall toxicity profile of the seeds. In our findings, squamocin was found to produce potent, multi-organ toxic effects at nanomolar concentrations, encompassing developmental teratogenicity, cartilage dysplasia, neurotoxicity, and metabolic perturbation.

In our investigation, Squamocin exhibited ultra-potent toxicity in zebrafish larvae, with adverse effects manifesting at nanomolar concentrations. Acute exposure caused concentration-dependent mortality (${\mathrm{LC}}_{10}$ 62 nM; ${\mathrm{LC}}_{50}$ 75 nM, Figure 2A). Beyond lethality, squamocin induced distinct developmental teratogenicity, including spinal curvature, pericardial edema, and delayed yolk sac absorption, with malformation rates increasing dose-dependently (Figure 2C). Importantly, these developmental disruptions occurred without substantial growth retardation, as larval body length remained largely unchanged (Figure 2D), indicating that squamocin selectively targets organogenesis rather than general somatic growth.

Concomitant with these developmental defects, squamocin severely impaired craniofacial cartilage morphogenesis. It disrupted the spatial architecture and polarized alignment of craniofacial cartilages, resulting in abnormal intercartilaginous connections, cranial base collapse, and facial asymmetry (Figure 3). Specifically, exposure shortened the interchondral distance between Meckel’s cartilage and the ceratohyal, reduced ceratohyal length, and enlarged the ceratohyal angle. These structural defects were accompanied by suppressed chondrocyte proliferation, differentiation, and maturation at the cellular level (Figure 3D), ultimately leading to chondrohypoplasia and disrupted craniofacial symmetry. Collectively, these defects contributed to the overall craniofacial malformation and teratogenic outcome.

### 3.3. Dose-Dependent Neurotoxicity and Locomotor Phenotype Switching

Neurotoxicity represents another prominent adverse effect of squamocin. At 20 nM, the compound significantly reduced total swimming distance and average speed (Figure 4A,B). This reduction reflected orderly locomotor suppression. Cholinergic inhibition and mild oxidative stress underpinned this effect, and body malformations remained mild. At 40 nM, however, both total swimming distance and average speed increased. This apparent recovery did not indicate reduced toxicity. Instead, severe body malformations (Figure 4), extensive neuronal apoptosis (Figure 4D), and elevated oxidative stress (Figure 5B) occurred concurrently. The expression of neuroinflammation-related genes (*rigi*, *pipox*, and *myd88*) also increased. These multi-system injuries severely disrupted motor coordination. The larvae lost directed swimming ability and exhibited disordered tremors and spasmodic struggling. Episodic abnormal activity offset the distance reduction caused by neural inhibition. Consequently, total swimming distance showed no apparent difference from the control group. These results indicate that 40 nM squamocin elicits more severe neurotoxicity than 20 nM. However, the phenotypic manifestation shifts from reduced locomotor output to disordered locomotor patterns.

Biochemically, squamocin potently inhibited AChE activity (Figure 5A) and induced dose-dependent ROS accumulation (Figure 5B,C), thereby disrupting cholinergic neurotransmission and triggering oxidative stress with consequent neural damage. Under acute toxicant exposure, reduced AChE activity is recognized as a direct driver of neuronal injury [16], and elevated ROS levels correlate positively with neuronal and oligodendrocyte loss, demyelination, glial scar formation, and motor dysfunction [17]. The concomitant disruption of cholinergic neurotransmission and induction of oxidative stress thus point to a dual mechanism underlying squamocin-induced neural damage. Fine-scale behavioral parameters in zebrafish warrant further investigation.

The AchE assay measured total cholinesterase activity without selective inhibitors for AChE or butyrylcholinesterase (BuChE). Consequently, the observed inhibition may reflect reduced activity of AChE, BuChE, or both. In the healthy brain, AChE is the principal enzyme for acetylcholine hydrolysis, while BuChE serves only a minor regulatory role [18]. Inhibition of either enzyme slows acetylcholine degradation, resulting in elevated local acetylcholine concentrations [19]. At concentrations as low as 10 and 20 nM, squamocin significantly decreased AChE activity (Figure 5A). Furthermore, the neurotoxicity can also be found by multiple independent lines of evidence, including impaired locomotor behavior, neuronal apoptosis, and elevated ROS. The cholinesterase inhibition data thus represent one component of a converging evidence chain, and the robustness of the overall conclusion is maintained even when accounting for this assay limitation.

### 3.4. Squamocin Disrupts Amino Acid Metabolism and Stress-Related Signaling

Squamocin markedly disrupted metabolic homeostasis in zebrafish larvae, triggering widespread alterations in endogenous metabolite levels (Figure 6). Enrichment analysis revealed that disturbed metabolites converged on specific pathways (Figure 7), and the altered expression of *pipox* and *myd88* further corroborated these results, confirming that squamocin perturbed lysine degradation (dre00310), glycan degradation (dre00510), and the C-type lectin receptor signaling pathway (dre04625) (Figure 8).

The integration of untargeted metabolomics and qRT-PCR data indicates that squamocin exerts its toxic effects, at least in part, through concurrent disruption of amino acid metabolism and glycan processing. Lysine degradation (dre00310) emerged as one of the most significantly affected pathways. As an essential amino acid required for energy metabolism and protein synthesis, impaired lysine degradation can compromise embryonic development. Previous reports [20] in Nile tilapia have linked lysine metabolic disturbance to adverse physiological outcomes, highlighting the broad developmental consequences of lysine imbalance. Glycine metabolism also appears relevant. Elevated glycine levels can induce lysine-glycylation, thereby suppressing TEK-PI3K-AKT/FOXO1 signaling and producing teratogenic effects [21]. In addition, as an inhibitory neurotransmitter, glycine imbalance may disrupt glycinergic neurotransmission and impair motor coordination, analogous to the scoliosis phenotype observed in *slc6a9*-mutant zebrafish [22].

In addition to amino acid metabolism, the C-type lectin receptor signaling pathway (dre04625) and the RIG-I-like/Toll-like receptor axes are implicated in the toxic response. C-type lectin receptors participate in trained immunity regulation and inflammatory control [23], whereas aberrant activation of RIG-I-like and Toll-like receptors can drive neuroinflammation, oxidative stress, and apoptosis [23].

Collectively, these metabolic and transcriptional alterations provide a mechanistic framework that reconciles the observed developmental teratogenicity, neurotoxicity, and lethal effects of squamocin. By disrupting amino acid metabolism and glycan processing while concurrently perturbing stress-related signaling pathways, squamocin converges on impaired organogenesis and neural function.

## 4. Materials and Methods

### 4.1. Chemicals and Materials

Paraformaldehyde, PBS, Dimethyl sulfoxide (DMSO), and propylene glycol were purchased from Solarbio (Solarbio Life Sciences Co., Ltd., Beijing, China). AG RNAex Pro RNA reagent, Evo M-MLV kit, and Premix Pro Taq HS qPCR kit were purchased from Accurate (Accurate Biotechnology Co., Ltd., Changsha, China). The primers (Table S2 in Supplementary Material) were obtained from Sangon (Sangon Biotech Co., Ltd., Shanghai, China).

### 4.2. Zebrafish Toxicity-Guided Isolation of Squamocin

Dried seeds of *A. squamosa* (1 kg, collected from Guangxi, China) were powdered and extracted with ethanol (10 L) under ultrasonication for 1 h. The extraction was repeated twice. The extracts were filtered, combined, and concentrated under reduced pressure to yield the crude ethanol extract. The crude extract was suspended in water and successively partitioned with equal volumes of petroleum ether and ethyl acetate to afford the petroleum ether fraction, ethyl acetate (EtOAc) fraction, and aqueous residue.

Each fraction and the aqueous residue were dissolved in DMSO and diluted with E3 medium to a test concentration of 0.7 µg/mL. Zebrafish larvae at 2 days post-fertilization (dpf) were exposed to each sample (30 larvae per group), and mortality was recorded as the toxicity indicator. The EtOAc fraction exhibited the highest toxicity and was subsequently separated by silica gel column chromatography (200–300 mesh) using a gradient elution of dichloromethane/methanol (100:1, 20:1, and finally pure methanol) to yield four subfractions (Fr.1–Fr.4).

Fr.2, which showed the highest toxicity, was further purified by preparative high-performance liquid chromatography (prep-HPLC) on a YMC-Pack ODS-A column (250 mm × 10.0 mm i.d., 5 µm) at a flow rate of 2.0 mL/min, with an acetonitrile/water gradient (0–20 min, 90% acetonitrile; 20–40 min, 100% acetonitrile). Fractions were collected every 2 min, yielding 20 tubes (Fr.2.2.1–Fr.2.2.20). Toxicity assessment identified Fr.2.2.1–Fr.2.2.4 as toxic fractions, whereas the remaining fractions were inactive.

The isolation workflow is illustrated in Figure 1. The most toxic fraction, Fr.2.2.2, was further purified by HPLC on a YMC-Pack ODS-A column (250 mm × 10.0 mm i.d., 5 µm particle size, 12 nm pore size; YMC Co., Ltd., Kyoto, Japan) eluted with 70–100% MeCN (2 mL/min) to afford squamocin (47.1 mg). Quantitative analysis of squamocin in the seeds was performed by LC-MS using an external standard method. For details of the LC-MS analysis, see Section 4.11.

**Squamocin**, oil. H1 NMR (400 MHz): δ 0.88 (3H, t, *J* = 6.7 Hz), 1.36(3H, d, *J* = 6.8 Hz), 1.30–1.60 (41H, m), 1.61–1.70 (3H, m), 1.90–2.00 (4H, m), 2.21 (2H, tt, *J* = 7.9, 1.5 Hz), 3.33 (1H, dt, *J* = 10, 8.0 Hz), 3.38 (1H, m), 3.52(1H, m), 3.58(1H, m), 3.85 (4H, m), 5.05qq (1H, *J* = 6.8, 1.5 Hz), 7.20 (1H, q, *J* = 1.5 Hz). C13 NMR (100 MHz): δ 14.10, 19.21, 22.03, 22.63, 24.80, 25.16, 25.64, 25.67, 27.38, 28.45, 28.95, 29.04, 29.06, 29.18, 29.31, 29.39, 29.51, 29.61, 29.75, 29.85, 31.86, 32.40, 33.15, 37.24, 37.47, 40.94, 71.36, 71.73, 74.23, 77.45, 82.20, 82.58, 82.83, 83.37, 134.29, 148.93, 173.96. HR-ESI-MS: *m*/*z* full ${\mathrm{MS [M+H]}}^{+}$, found 623.4868, calcd. 623.48813 (error 2.13 ppm). The H1 and C13 NMR and HRMS spectra were consistent with reported data [9].

### 4.3. Zebrafish Maintenance

The adult zebrafish were procured from EzeRinka (EzeRinka Biotechnology Co., Ltd., Nanjing, China). Zebrafish (*Danio rerio*) embryos were maintained in E3 medium at 28 °C under a 14/10 h light/dark cycle [24].

### 4.4. Acute Toxicity Assay

At 2 dpf, larvae were distributed into six-well plates (20 larvae per well, three replicates per group) and exposed to serial concentrations of squamocin (20–100 nM) for 48 h at 28 °C, with medium renewal every 24 h. Mortality was recorded at the end of exposure, with death defined as cessation of heartbeat, developmental arrest, or embryo coagulation. The rate was calculated as the percentage of dead larvae relative to the total number of exposed larvae [25].

### 4.5. Developmental Toxicity Assay

The procedures were performed according to previously reported methods with minor modifications [26]. At 1 day post-fertilization (dpf), embryos were exposed to 20 or 40 nM squamocin until 5 dpf (20 embryos per group, three replicates). The hatching rate was recorded at 2 dpf. At 5 dpf, morphological abnormalities, including spinal curvature, pericardial edema, and delayed yolk sac absorption, were examined and documented under a stereomicroscope (Nikon SMZ25, Tokyo, Japan). Body length was measured in nine randomly selected larvae per group as the distance from the anterior tip of the head to the end of the caudal fin, using captured images analyzed with ImageJ (1.54) software (National Institutes of Health, Bethesda, MD, USA). The hatching rate was calculated as the percentage of hatched larvae relative to the total number of exposed embryos. The malformation rate was calculated as the percentage of malformed larvae relative to the total number of surviving larvae.

### 4.6. Cartilage Staining and Analysis

The procedures were adapted from previously reported methods [27,28] with minor modifications. At 2 dpf, larvae were exposed to 0.1% DMSO, 20 nM, or 40 nM squamocin until 5 dpf (10 larvae per well, three wells per group). At 5 dpf, larvae were fixed in 4% paraformaldehyde for 4 h, washed three times with PBS, and bleached in 3% $H_{2}$$O_{2}$/0.5% KOH. After further PBS washes, specimens were stained with 0.1% Alcian blue in the dark for 12 h. Samples were dehydrated through acidified ethanol (70% ethanol with 5% HCl), rehydrated sequentially in 75%, 50%, and 25% acidified ethanol, washed with PBS, and stored in glycerol. Craniofacial cartilages were visualized under a stereomicroscope (Nikon SMZ25). The interchondral distance between Meckel’s cartilage and the ceratohyal, the length of the ceratohyal, and the corresponding angle were measured using ImageJ.

### 4.7. Locomotor Behavior Analysis

At 2 dpf, larvae were exposed to 20 nM or 40 nM squamocin until 5 dpf (20 larvae per group, three replicates) [29]. At 5 dpf, larvae were washed three times with E3 medium. Nine larvae per group were transferred to a six-well plate (3 mL E3 medium per well), acclimatized for 10 min under a video-equipped microscope (BC4K-36D2), and locomotor trajectories were recorded for 30 min. Total locomotor distance and average swimming speed were analyzed using EthoVision XT 8.0 software (Noldus Information Technology, Wageningen, The Netherlands).

### 4.8. Neuronal Apoptosis Detection

Neuronal apoptosis was assessed by acridine orange (AO) staining [30]. Following exposure (2–5 dpf), larvae were washed three times with PBS and incubated in E3 medium containing 5 mg/L AO at 28 ± 0.5 °C in the dark for 30 min. After three additional PBS washes, larvae were anesthetized with 0.04% tetracaine and examined under a stereomicroscope (Nikon SMZ25). Apoptotic cells in the brain were identified as bright green fluorescent puncta. Fluorescence intensity was quantified using ImageJ.

### 4.9. ROS Level Measurement

Reactive oxygen species (ROS) were measured using 2′,7′-dichlorodihydrofluorescein diacetate (DCFH-DA) [30]. At 5 dpf, exposed larvae were incubated in E3 medium containing 20 µg/mL DCFH-DA at 28 °C in the dark for 60 min. After washing and anesthesia, fluorescence in the brain region was captured under a stereomicroscope (Nikon SMZ25). Mean fluorescence intensity was quantified using ImageJ.

### 4.10. Acetylcholinesterase (AChE) Activity Assay

AChE activity was determined using an AChE assay kit (Solarbio Life Sciences Co., Ltd., Beijing, China). The assay was performed according to the manufacturer’s instructions and the reported method [31]. Briefly, for each concentration group, four replicates were prepared, and 65 larvae were randomly selected per replicate. The larvae were centrifuged and weighed, then homogenized on ice in extraction buffer at a solid-to-liquid ratio of 1:10 (g/mL). The homogenate was centrifuged at 8000× *g* for 10 min at 4 °C, and the resulting supernatant (30 µL) was mixed with 100 µL of Reagent II (substrate) and incubated in a 37 °C water bath for exactly 5 min. Subsequently, 100 µL of Reagent IV (chromogenic reagent) was added, and the mixture was vortexed and centrifuged at 12,000 rpm for 5 min at room temperature. Then, 50 µL of the supernatant was transferred to a new tube, mixed with 850 µL of Reagent I and 100 µL of Reagent III, and allowed to stand for 2 min. The absorbance at 412 nm was measured using a UV-1780 visible spectrophotometer (Shimadzu, Kyoto, Japan) and recorded as the assay absorbance ($A_{\mathrm{sample}}$). For the control tube, Reagent I (100 µL) was substituted for Reagent II, and the same procedure was followed to obtain the control absorbance. The difference in absorbance and AChE activity were calculated according to the following equations:

(1) $$\mathrm{AChE}\;\mathrm{activity}\;\left(U/g\right)=\frac{2255\times (A_{\mathrm{sample}}-A_{\mathrm{control}})}{W}$$

where *W* is the sample weight (g). One unit (U) of AChE activity was defined as the amount of enzyme required to catalyze the formation of 1 nmol of TNB per minute per gram of tissue.

### 4.11. Metabolomic Sample Collection, Detection, and Data Analysis

The procedures were adapted from reported protocols with minor modifications [32]. At 2 dpf, zebrafish larvae were randomly divided into a blank control group (G0) and squamocin-exposed groups (G1 and G2). Each group consisted of seven biological replicates, with 75 larvae per replicate. Following 3 days of exposure, larvae were washed three times with E3 medium, immediately snap-frozen in liquid nitrogen, and lyophilized using a freeze dryer. The lyophilized samples were accurately weighed, and metabolites were extracted in 80% methanol at a solid-to-liquid ratio of 1:10 (*w*/*v*) by ultrasonication in an ice bath using a cell disruptor. The extracts were then centrifuged at 12,000 r/min for 20 min at 4 °C, and the resulting supernatants were collected for LC–MS/MS analysis.

Untargeted metabolomic profiling was performed on an UHPLC-Q-Orbitrap system (Thermo Q Exactive Focus LC-MS system, Thermo Fisher Scientific Inc., Waltham, MA, USA) operating in both positive and negative electrospray ionization (ESI) modes. Chromatographic separation was achieved on a reversed-phase $C_{18}$ column with polar endcapped (Thermo Syncronics 1.7 µm, 100×2.1 mm) at 30 °C with a flow rate of 0.3 mL/min. The mobile phase contained 0.1% acetic acid, with acetonitrile increasing linearly from 1% to 100% over 15 min. The mass spectrometer was operated with a mass range of *m*/*z* 70–1000, and the source parameters were set as follows: capillary voltage, 3.0 kV (positive)/2.5 kV (negative); desolvation temperature, 350 °C; source temperature, 100 °C; collision energy (CE), 10, 20 and 40 V.

Raw MS data were processed using MS-Dial (version 5.5) for peak detection, retention time correction, and peak alignment. Metabolite annotation was performed by matching the accurate mass and tandem MS/MS spectra against the KEGG databases [33] with a mass tolerance of 0.003 and 0.005 *m*/*z*. Data normalization was performed using total ion current (TIC) normalization. Principal component analysis (PCA) was conducted to visualize the overall metabolic separation among groups. Differential metabolites were identified by Spearman correlation analysis across the concentration gradient (G0 → G1 → G2), with the Spearman test. Pathway enrichment analysis of the significantly altered metabolites was performed using the FELLA package in R (version 4.2.0) [34] based on the KEGG database of *Danio rerio*, with significance determined by permutation testing (p<0.01).

### 4.12. RT-qPCR

Total RNA was extracted from zebrafish larvae using AG RNAex Pro RNA reagent (Accurate Biotechnology Co., Ltd., Changsha, China) according to the manufacturer’s protocol and the reported method [35]. Briefly, 15 larvae per group were homogenized in 1 mL RNAex Pro reagent, and total RNA was purified by chloroform extraction and isopropanol precipitation. The cDNA was synthesized using the Evo M-MLV RT Kit (Accurate Biotechnology) with oligo(dT) primers. Quantition was carried out on a CFX96 Touch Real-Time PCR Detection (Bio-Rad, Singapore), using the Premix Pro Taq HS qPCR Kit (Accurate Biotechnology). Relative mRNA expression levels were calculated using the $2^{-\Delta \Delta C_{t}}$ method, and each sample was analyzed in triplicate.

### 4.13. Data Analysis

All data visualization and statistical analyses were performed in Python (version 3.13) using pandas (version 2.0), plotnine (version 0.15) and igraph (version 1.0). Two-group comparisons were evaluated by Student’s *t*-test, whereas multiple groups were assessed by one-way analysis of variance (ANOVA). Statistical significance was set at p<0.05.

## 5. Conclusions

Through a toxicity-guided isolation strategy using zebrafish larvae, squamocin was identified as the principal toxic constituent of *A. squamosa* seeds. To our knowledge, this represents the first identification of an acute toxic constituent from this plant. In zebrafish larvae, squamocin exerted potent, multi-organ toxicity at nanomolar concentrations, encompassing acute lethality, developmental teratogenicity, and craniofacial cartilage dysplasia. Notably, these developmental disruptions occurred without substantial growth retardation, indicating selective targeting of organogenesis rather than general somatic growth. Concurrently, squamocin induced neurotoxicity through a dual mechanism involving acetylcholinesterase inhibition and reactive oxygen species accumulation, leading to locomotor impairment and neuronal apoptosis. Untargeted metabolomics further revealed that squamocin exposure disturbed metabolic homeostasis, with altered metabolites converging on lysine degradation, glycan degradation, and stress-related signaling pathways including C-type lectin receptor and RIG-I-like receptor axes. The qRT–PCR validation confirmed altered expression of *pipox* and *myd88*, supporting the perturbation of amino acid metabolism and stress-related signaling. Collectively, these findings establish squamocin as the major toxic constituent of *A. squamosa* seeds and provide a mechanistic framework for its broad toxicological spectrum, offering new scientific guidance for the safe and rational use of this material in traditional medicine and food.

## Data Availability

Data are contained within the article.

## Supplementary Materials

The following supporting information can be downloaded at: https://www.mdpi.com/article/10.3390/ijms27156802/s1.

## Abbreviations

The following abbreviations are used in this manuscript:

ACGs	annonaceous acetogenins
AChE	acetylcholinesterase
ANOVA	analysis of variance
AO	acridine orange
CAP	Caribbean atypical parkinsonism
DCFH-DA	$2^{\prime }$,$7^{\prime }$-dichlorodihydrofluorescein diacetate
DMSO	dimethyl sulfoxide
dpf	days post-fertilization
ESI	electrospray ionization
HR-ESI-MS	high-resolution electrospray ionization mass spectrometry
HSP90α	heat shock protein 90α
KEGG	Kyoto Encyclopedia of Genes and Genomes
NMR	nuclear magnetic resonance
PCA	principal component analysis
PBS	phosphate-buffered saline
qRT-PCR	quantitative real-time PCR
ROS	reactive oxygen species
TIC	total ion current
UHPLC	ultra-high-performance liquid chromatography
