# Supplementary material for: Squamocin as the Principal Toxic Constituent of Annona squamosa Seeds: Toxicity-Guided Isolation, Developmental and Neurotoxicity, and Metabolic Perturbations
Source: Int J Mol Sci. 2026 Jul 29;27(15):6802. doi: 10.3390/ijms27156802 (PMC13466299; doi:10.3390/ijms27156802)
Supplement: Supplementary file 1 [file ijms-27-06802-s001.zip › ijms-4409318-supplementary.pdf]

Supplementary materials

Toxicity-Guided Isolation of Squamocin from *Annona squamosa* Seeds: Developmental Toxicity and In Vivo Metabolic Perturbations

Chenyuan Qi, Lu Zhang, Xue Li, Mengling Zhang, Disheng Wang, and Qiang Zhang *

Shaanxi Key Laboratory of Natural Products & Chemical Biology, College of Chemistry & Pharmacy, Northwest A&F University, Yangling 712100, China.

*Corresponding author, Qiang Zhang, Email: zhangq@nwsuaf.edu.cn

Contents

Figure S1. ^{1}H (400 MHz) ,^{13}C (100 MHz) NMR and LC-MS/MS spectrum of Squamocin....................................2

Table S1. Metabolic enrichment results based on Fella (p < 0.01) 6

Table S2. Primers used for qPCR analysis....................................8

Figure S1. ^{1}H (400 MHz) ,^{13}C (100 MHz) NMR and LC-MS/MS spectrum of Squamocin.

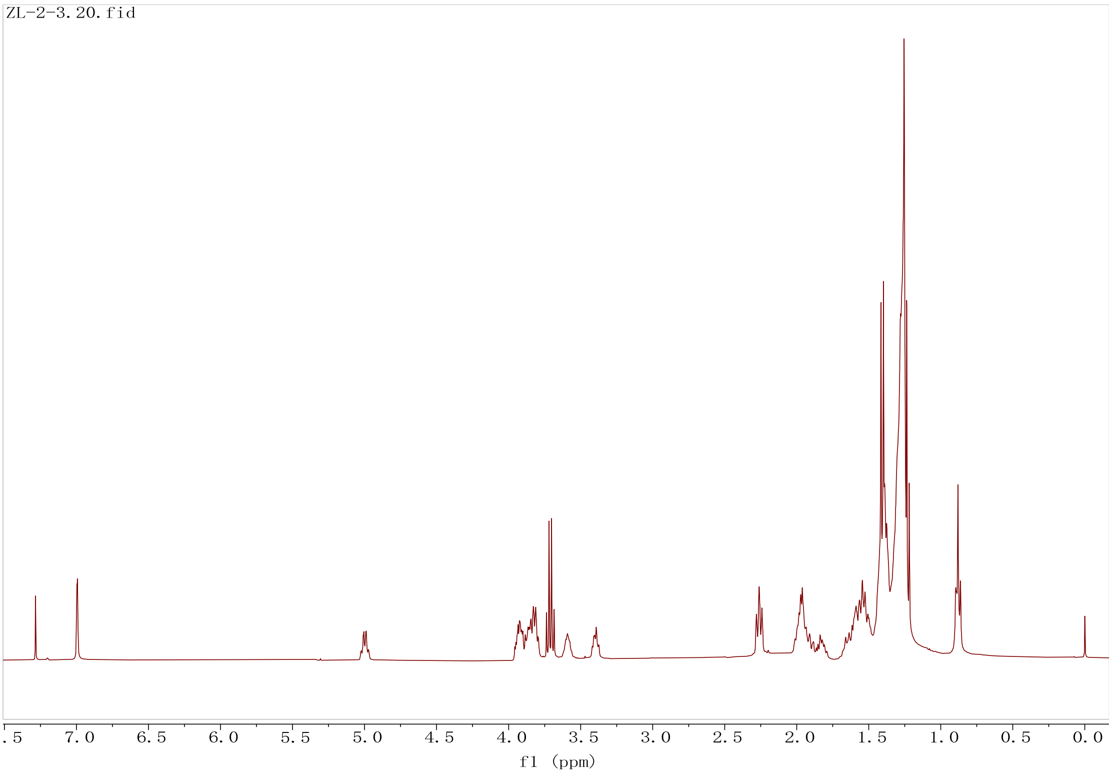

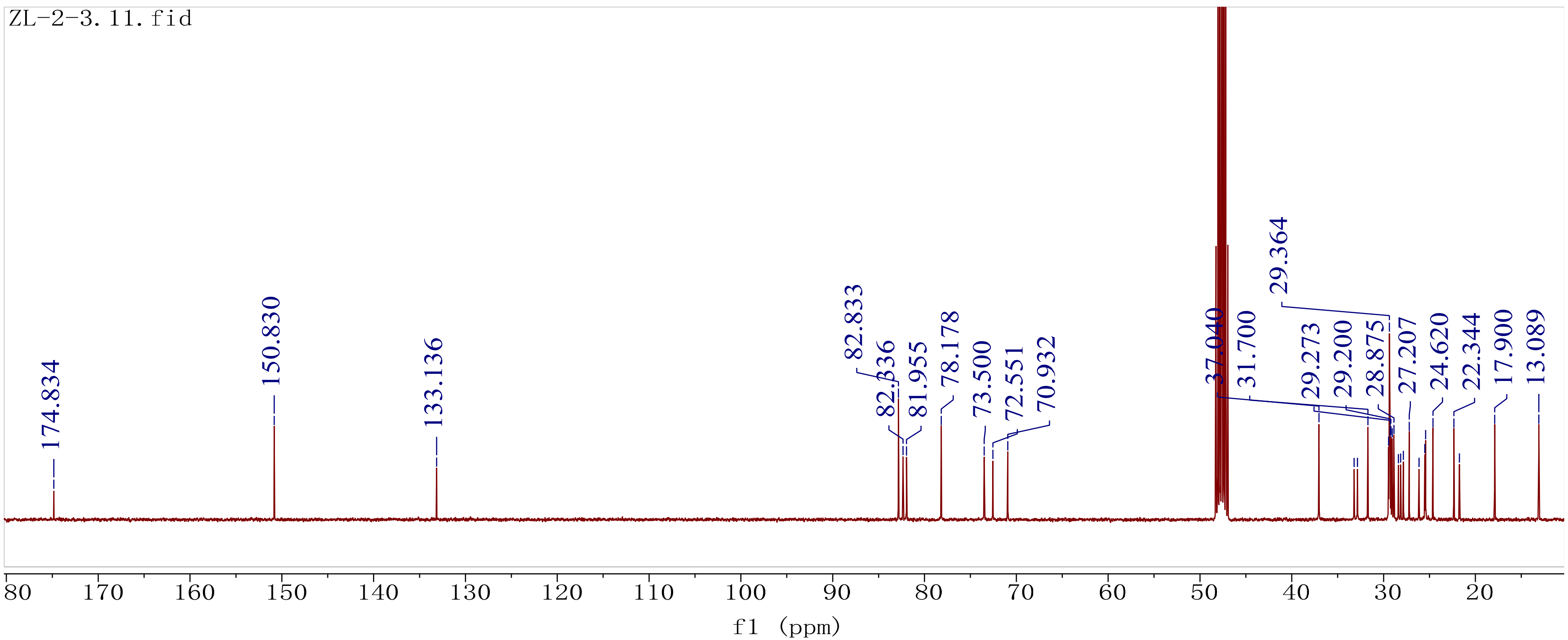
ZL-2-3.11.fid
174.834
150.830
133.136
82.833
82.336
81.955
78.178
73.500
72.551
70.932
37.040
31.700
29.364
29.273
29.200
28.875
27.207
24.620
22.344
17.900
13.089
80
170
160
150
140
130
120
110
100
90
80
70
60
50
40
30
20
f1 (ppm)

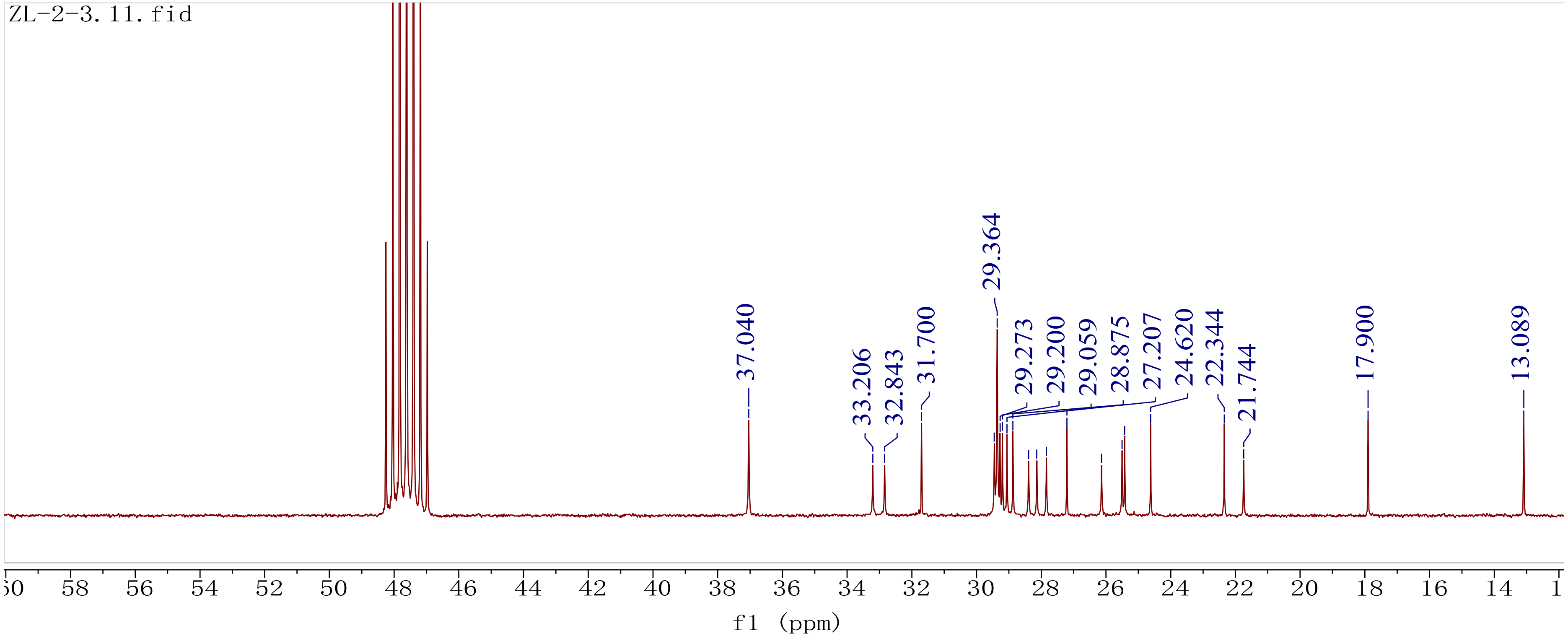
ZL-2-3.11.fid
37.040
33.206
32.843
31.700
29.364
29.273
29.200
29.059
28.875
27.207
24.620
22.344
21.744
17.900
13.089
f1 (ppm)

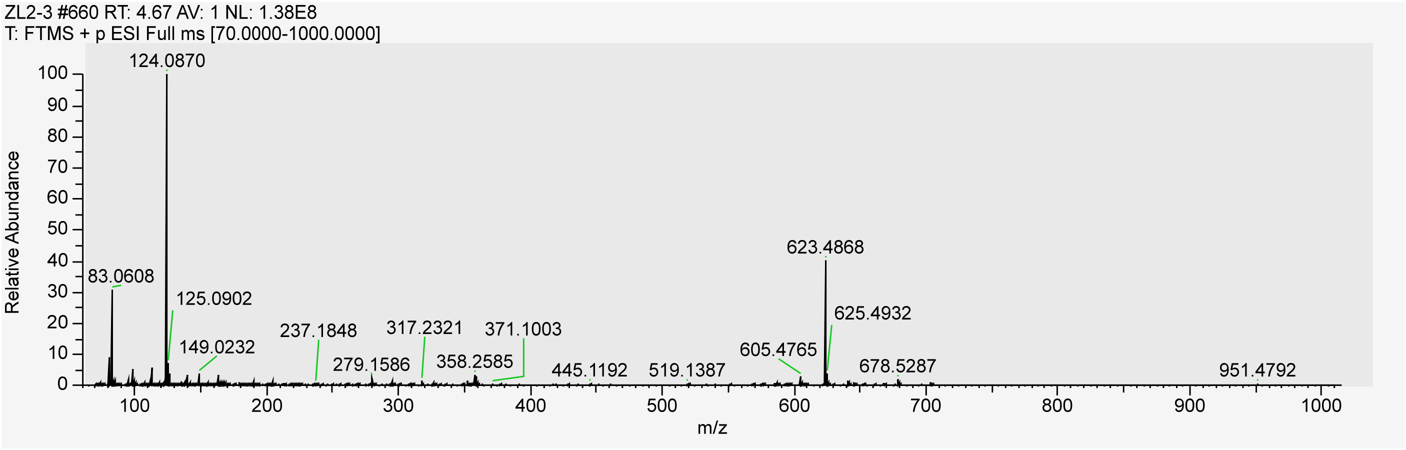

HR-ESI-MS m/z full MS [M+H]+, calcd. 623.48813, found 623.4868 (error 2.13 ppm).

Table S1. Metabolic enrichment results based on Fella ($p < 0.01$)

KEGG ID	Type	Name	p value
dre00310	pathway	Lysine degradation	0.000001
dre04625	pathway	C-type lectin receptor signaling pathway	0.000001
dre00511	pathway	Other glycan degradation	0.000004
dre00260	pathway	Glycine, serine and threonine metabolism	0.000073
dre04142	pathway	Lysosome biogenesis	0.010110
dre04622	pathway	RIG-I-like receptor signaling pathway	0.036681
dre04620	pathway	Toll-like receptor signaling pathway	0.040044
M00974	module	Betaine metabolism, animals, betaine => glycine	0.000009
M01001	module	GDP-L-Fuc biosynthesis, L-Fuc => GDP-L-Fuc	0.018510
1.1.1.122	enzyme	D-threo-aldose 1-dehydrogenase	0.000001
1.5.3.7	enzyme	L-pipecolate oxidase	0.000001
3.2.1.51	enzyme	alpha-L-fucosidase	0.000001
3.2.1.31	enzyme	beta-glucuronidase	0.000001
3.2.1.96	enzyme	mannosyl-glycoprotein endo-beta-N-acetylgluco...	0.000004
1.5.3.1	enzyme	sarcosine oxidase (formaldehyde-forming)	0.000076
3.4.22.61	enzyme	caspase-8	0.000079
3.6.1.7	enzyme	acylphosphatase	0.000236
3.4.19.12	enzyme	ubiquitinyl hydrolase 1	0.001961
2.7.1.52	enzyme	fucokinase	0.004230
3.4.22.38	enzyme	cathepsin K	0.007897
3.5.1.26	enzyme	N4-(beta-N-acetylglucosaminyl)-L-asparaginase	0.011376
3.4.22.42	enzyme	cathepsin O	0.012238
2.1.1.361	enzyme	[histone H4]-lysine20 N-methyltransferase	0.012826
2.7.11.10	enzyme	IkappaB kinase	0.036687
C00408	compound	L-Pipecolate	0.000001
C00180	compound	Benzoate	0.000001
C00450	compound	(S)-2,3,4,5-Tetrahydropyridine-2-carboxylate	0.005193
C00191	compound	D-Glucuronate	0.000001
C06231	compound	Ectoine	0.000001
C00423	compound	trans-Cinnamate	0.000001
C00489	compound	Glutarate	0.000001
C01019	compound	L-Fucose	0.000001
R01426	reaction	Benzoate + Acetate + NADH + H+ <=> trans-Cinn...	0.000001
R02401	reaction	glutarate-semialdehyde:NAD+ oxidoreductase	0.000001
R12216	reaction	glutarate,2-oxoglutarate:oxygen oxidoreductas...	0.000001
R13035	reaction	Glutarate + Succinyl-CoA <=> Glutaryl-CoA + S...	0.000001
R02402	reaction	glutarate:CoA ligase (ADP-forming)	0.000001
R03161	reaction	ATP:L-fucose 1-phosphotransferase	0.000001
R02205	reaction	L-pipecolate:(acceptor) 1,6-oxidoreductase	0.000001
R08414	reaction	(S)-2,3,4,5-Tetrahydropyridine-2-carboxylate ...	0.000001
R02201	reaction	L-pipecolate:NAD+ 2-oxidoreductase	0.000001
R02203	reaction	L-pipecolate:NADP+ 2-oxidoreductase	0.000001

R09800	reaction	ectoine aminohydrolase	0.000001
R08926	reaction	L-fucose:NAD+ 1-oxidoreductase	0.000001
R08050	reaction	Ectoine + 2-Oxoglutarate + Oxygen <=> 5-Hydro...	0.000001
R02204	reaction	L-pipecolate:oxygen 1,6-oxidoreductase	0.000001
R06979	reaction	ectoine hydro-lyase	0.000001
R04979	reaction	bilirubin beta-diglucuronide glucuronosohydro...	0.001713
R10830	reaction	beta-D-glucuronoside glucuronosohydrolase	0.000001
R08260	reaction	SN38 glucuronide glucuronosohydrolase	0.002518
R07818	reaction	G13040 + H2O <=> G09660 + D-Glucuronate	0.000001
R01478	reaction	beta-D-glucuronoside glucuronosohydrolase	0.000139

Table S2. Primers used for qPCR analysis

Gene Symbol	Oligo Name	Sequence (5' to 3')
ctsl	ctsl-F	GACTGTTCCAGACCACAGGG
	ctsl-R	TACCGGCATGGCAGATCATC
pipox	pgls-F	AAGCTGAAGGACACGATGCA
	pgls-R	GCACAGCTTGGAGGGATCTT
ctsd	h6pd-F	GCACTCCTGTCCAGACCTTC
	h6pd-R	GATGAAGCAAGCAGGCGATG
nlrp3	nlrp3-F	CTCGTGCCAGCCGTGAGATG
	nlrp3-R	CGCCTCCAGACTGTAGTTGTAGC
aass	aass-F	GGACCCTGGCATTGATCACA
	aass-R	GTGTTGAGCAGGACACCGTA
tlr4ba	tlr4ba -F	GGGAAGTCAATCGCCTCCAA
	tlr4ba -R	GTTGGCATTGCGTTCCATCA
rigi	rigi-F	AAAACCAGATGGAGGCGGAG
	rigi-R	CGTATGACCTGAGCTCGTCC
myd88	myd88-F	TTGACGGACTGGGAAACTCG
	myd88-R	CCTGAAGCGGTTTCCTCTGT
aldh7a1	aldh7a1-F	CACCAAACAGGCTGTTCAGC
	aldh7a1-R	CATAGTTACCCGGCCGATCC
map1lc3b	map1lc3b-F	AAGGGAGAGAAGCAACTGCC
	map1lc3b-R	GACCAGCAGGAAGAAAGCCT
gusb	gusb-F	TTCCACGGGGTCAACAAACA
	gusb-R	GCAGGATCTCCTCAGCGTAC
tnfa	tnfa-F	ACATCAGCTGCACGTCTGAA
	tnfa-R	CGTGCAGATTGAGCGGATTG
β-actin	β-actin-F	AACGAACGACCAACCTAAACCTCTC
	β-actin-R	CTCCCTTTCCAGTTTCCGCATCC
